# Longitudinal immunosequencing in healthy people reveals persistent T cell receptors rich in highly public receptors

**DOI:** 10.1186/s12865-019-0300-5

**Published:** 2019-06-21

**Authors:** Nathaniel D. Chu, Haixin Sarah Bi, Ryan O. Emerson, Anna M. Sherwood, Michael E. Birnbaum, Harlan S. Robins, Eric J. Alm

**Affiliations:** 1 Department of Biological Engineering, Massachusetts Institute of Technology, Cambridge, MA USA; 2 Microbiology Graduate Program, Massachusetts Institute of Technology, Cambridge, MA USA; 3 Center for Microbiome Informatics and Therapeutics, Massachusetts Institute of Technology, Cambridge, MA USA; 4 Computational and Systems Biology Program, Massachusetts Institute of Technology, Cambridge, MA USA; 5 Adaptive Biotechnologies, Seattle, WA USA; 6 Fred Hutchinson Cancer Research Center, Seattle, Washington USA; 7 Broad Institute, Cambridge, MA 02139 USA

**Keywords:** T cell receptor, Persistent receptors, Immunosequencing, Repertoire sequencing, Healthy controls, Memory T cell, Naive T cell, Public receptors

## Abstract

**Background:**

The adaptive immune system maintains a diversity of T cells capable of recognizing a broad array of antigens. Each T cell’s specificity for antigens is determined by its T cell receptors (TCRs), which together across all T cells form a repertoire of millions of unique receptors in each individual. Although many studies have examined how TCR repertoires change in response to disease or drugs, few have explored the temporal dynamics of the TCR repertoire in healthy individuals.

**Results:**

Here we report immunosequencing of TCR β chains (TCRβ) from the blood of three healthy individuals at eight time points over one year. TCRβ repertoires of all peripheral-blood T cells and sorted memory T cells clustered clearly by individual, systematically demonstrating that TCRβ repertoires are specific to individuals across time. This individuality was absent from TCRβs from naive T cells, suggesting that the differences resulted from an individual’s antigen exposure history, not genetic background. Many characteristics of the TCRβ repertoire (e.g., diversity, clonality) were stable across time, although we found evidence of T cell expansion dynamics even within healthy individuals. We further identified a subset of “persistent” TCRβs present across all time points. These receptors were rich in clonal and highly public receptors and may play a key role in immune system maintenance.

**Conclusions:**

Our results highlight the importance of longitudinal sampling of the immune system, providing a much-needed baseline for TCRβ dynamics in healthy individuals. Such a baseline will improve interpretation of changes in the TCRβ repertoire during disease or treatment.

**Electronic supplementary material:**

The online version of this article (10.1186/s12865-019-0300-5) contains supplementary material, which is available to authorized users.

## Background

T cells play a vital role in cell-mediated immunity, one branch of the adaptive immune response against foreign and self-antigens. Upon recognizing an antigen from an antigen-presenting cell, naive T cells activate and proliferate rapidly. This process stimulates an effector response to the immediate challenge, followed by generation of memory T cells, which form a lasting cohort capable of mounting more-efficient responses against subsequent challenges by the same antigen.

The key to the flexibility and specificity of T cell responses lies in the cells’ remarkable capacity to diversify their T cell receptor (TCR) sequences, which determine the antigens those cells will recognize. Most T cells display TCRs made up of two chains: an α and a β chain. Sequence diversity in these chains arises during T cell development, through recombination of three sets of gene segments: the variable (V), diversity (D), and joining (J) segments [1]. Random insertions and deletions at each genetic junction introduce still more diversity, resulting in a theoretical repertoire of 10^15^ unique receptors in humans [2]. Selective pressures during and after T cell development, as well as constraints on the number of T cells maintained by the body, limit this diversity to an observed 10^7^ (approximately) unique receptors per individual [2–5].

This TCR repertoire forms the foundation of the adaptive immune response, which dynamically responds to disease. Each immune challenge prompts expansions and contractions of different T cell populations, and new T cells are continually generated. Substantial research interest has focused on these dynamics in the context of immune system perturbations, including in cancer [6–9], infection [10, 11], autoimmune disorders [12, 13], and therapeutic trials [8, 14, 15]. Observing changes in TCR populations not only uncovers cellular mechanisms driving disease, but can inform development of new diagnostics, biomarkers, and therapeutics involving T cells.

Less research has explored TCR dynamics in healthy individuals. Previous studies found that some TCRs remain present in individuals over decades [16, 17], but these long-term studies may not directly relate to shorter-term events, such as diseases or treatments. Interpreting TCR dynamics when the immune system is challenged would be more straightforward if we had a clear picture of TCR dynamics in healthy individuals.

To help develop this picture, we report immunosequencing of peripheral TCR β chain (TCRβ) repertoires of three individuals at eight time points over 1 year. We focused on the TCRβ chain because, unlike the α chain, only one β chain can be expressed on each T cell [18], the β chain contains greater sequence diversity [19], and it more frequently interacts with presented antigens during recognition [20]. These factors suggest that TCRβ sequences should be sufficient to track individual T cells and their clones. Our analysis revealed overall individuality and temporal stability of the TCRβ pool. We also uncovered a set of temporally persistent TCRβs, which were more abundant, and shared across more people, than transitory TCRβs.

## Results

### T cell receptor repertoires show individuality and stability through time

**Fig. 1:**
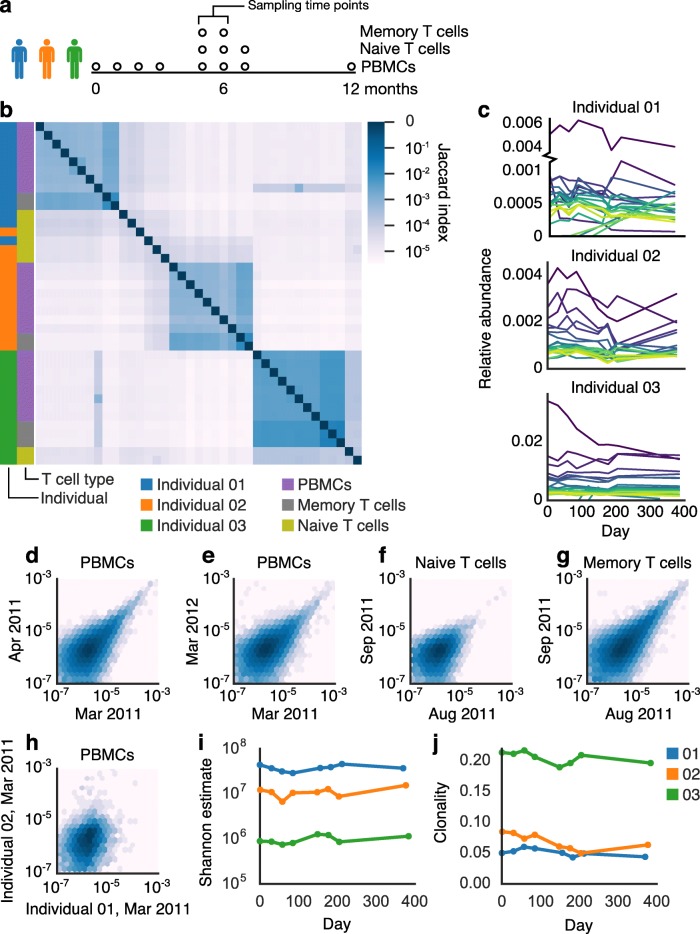
The TCRβ repertoire displayed stability and individual-specific characteristics across time. **a** Experimental design of T cell sampling. **b** A heatmap of Jaccard indexes shows clear clustering of samples by individual. Samples of naive T cells clustered less by individual than did PBMC or memory T cell samples. Relative abundances of the 20 most abundant TCRβs (**c**) appeared stable through time. TCRβ abundances in PBMCs correlated within an individual across time points, including across a month (**d**, shared TCRβs = 33,601, Spearman *rho* = 0.55718, *p* < 10^− 6^), and a year (**e**, shared TCRβs = 25,933, Spearman *rho* = 0.53810, *p* < 10^− 6^), as well as across a month in naive (**f**, shared TCRβs = 15,873, Spearman *rho* = 0.37892, *p* < 10^− 6^) and memory T cells (**g**, shared TCRβs = 47,866, Spearman *rho* = 0.64934, *p* < 10^− 6^). TCRβs correlated much less across individuals (**h**, shared TCRβs = 5014, Spearman *rho* = 0.28554, *p* < 10^− 6^). Shannon alpha diversity estimate (**i**) and clonality (defined as 1 – Pielou’s evenness, **j**) of the TCRβ repertoire were consistent over time

To characterize the dynamics of T cell receptors in healthy individuals, we deeply sequenced the TCRβ locus of all T cells from peripheral-blood mononuclear cells (PBMCs) isolated from three healthy adults (for schematic of experimental design, see Fig. 1a). We sampled each individual at eight time points over 1 year (Fig. 1a). For three intermediate time points, we also sequenced flow-sorted naive and memory T cells from PBMCs (see Methods). Our deep sequencing effort generated ~ 21 million (+/− 6 million SD) sequencing reads and ~ 250,000 (+/− 100,000 SD) unique, productive TCRβs—which we defined as a unique combination of a V segment, CDR3 amino acid sequence, and J segment [21]—per sample. These values and other summary statistics per sample appear in Additional file 2: Table S1. Most TCRβs had abundances near 10^− 6^ (Additional file 1: Figure S1), and rarefaction curves indicate that all samples were well saturated (Additional file 1: Figure S2). This saturation indicates that our sequencing captured the full diversity of TCRβs in our samples, although our blood samples cannot capture the full diversity of the TCRβ repertoire (see Discussion).

We first examined whether previously observed differences among individuals were stable through time [7, 22]. Looking at shared TCRβs (Jaccard index) among samples, we indeed found that samples of PBMCs or memory T cells taken from the same individual shared more TCRβs than samples taken from different individuals (Fig. 1b), and this pattern was consistent over one year. In adults, memory T cells are thought to make up 60–90% of circulating T cells [23, 24], which aligns with the agreement between these two T cell sample types. In contrast, TCRβs from naive T cells did not cluster cohesively by individual (Fig. 1b). As naive T cells have not yet recognized a corresponding antigen, this lack of cohesion might suggest one of two possibilities: (1) that before antigen recognition and proliferation, TCRβ repertoires are not specific to individuals or (2) the naive T repertoire is simply too diverse or too dynamic for individuality to manifest. We thus conclude that at the depth of sequencing and sampling of this study, individuality results from an individual’s unique antigen exposure and T cell activation history, which shape memory and total T cell repertoires.

We next examined patterns across samples from the same individual to understand TCR dynamics in healthy individuals. We observed only a minority of TCRβs shared among samples from month to month; indeed, samples of PBMCs at different months from the same individual typically shared only 11% of TCRβs (+/− 3.6% SD, range 5–18%) (Fig. 1b).

Two factors likely played a role in the observed turnover of TCRβ repertoires: (1) changes in TCRβ abundances in the blood across time and (2) inherent undersampling of such a diverse system (see Discussion). Surveying peripheral blood immune repertoires undersamples at multiple points, including blood drawing, nucleic acid extraction, library construction, and sequencing. The resulting undersampling likely explained much of the low overlap of TCRβs among samples but simultaneously highlighted the significance of TCRβs shared across time points. To verify that patterns we observed were not artifacts of undersampling, we also analyzed a subset of high-abundance TCRβs (those ranked in the top 1% by abundance, see Methods, Additional file 3), which are less likely to be affected. In these TCRβs, we observed typical sharing of 63% (+/− 13.8% SD, range 35–88%) of TCRβs in PBMC samples across time (Additional file 1: Figure S3a). PBMC and memory T cell samples (but not naive T cell samples) still clearly clustered by individual when only these TCRβs were considered (Additional file 1: Figure S3a).

The frequencies of high-abundance TCRβs from each individual were largely consistent over time (Fig. 1c). We found that abundances of the same TCRβs correlated within individuals over the span of a month (Fig. 1d, Additional file 1: Figure S3b) and a year (Fig. 1e, Additional file 1: Figure S3c). This correlation was particularly strong for abundant TCRβs (Additional file 1: Figure S3b–c) whereas rare TCRβs varied more. This correlation held true in naive and memory T cell subpopulations, sampled across a month (Fig. 1f-g). In contrast, correlation was much weaker among abundances of TCRβs shared across individuals (Fig. 1h, Additional file 1: Figure S3d), again highlighting the individuality of each repertoire. We found that the proportion of shared TCRβs (Jaccard index) tended to decrease with longer time intervals passed between samples, although with a notable reversion in Individual 02 (Additional file 1: Figure S4). We observed stable diversity (Fig. 1i, Additional file 1: Figure S3e), clonality (Fig. 1j, Additional file 1: Figure S3f), and V and J usage (Additional file 1: Figures S5, S6; Additional file 2: Tables S2 and S3) within individuals over time.

In the absence of experimental intervention, we observed complex clonal dynamics in many TCRβs, including cohorts of TCRβs with closely correlated expansion patterns (Additional file 1: Figure S7). To avoid artifacts from undersampling, we looked for such cohorts of correlating receptors only in high-abundance TCRβs (see Methods). In all individuals, many high-abundance TCRβs appeared together only at a single time point. We also found cohorts of high-abundance TCRβs that correlated across time points (Additional file 1: Figure S7). Some of these cohorts included TCRβs that fell across a range of abundances (Additional file 1: Figure S7a-b), while other cohorts were made up of TCRβs with nearly identical abundances (Additional file 1: Figure S7c). Correlating TCRβs were not obviously sequencing artifacts (Additional file 2: Table S4, Methods). These cohorts of closely correlated TCRβs indicate that even in healthy individuals whose overall TCR repertoire appears stable, underlying dynamics remain.

Taken together, these results revealed a diverse system, which nevertheless displayed consistent, unifying features differentiating individuals, plus longitudinal dynamics that suggested continual immune processes.

### A persistent TCRβ repertoire contains elevated proportions of clonal, highly public TCRβs

**Fig. 2:**
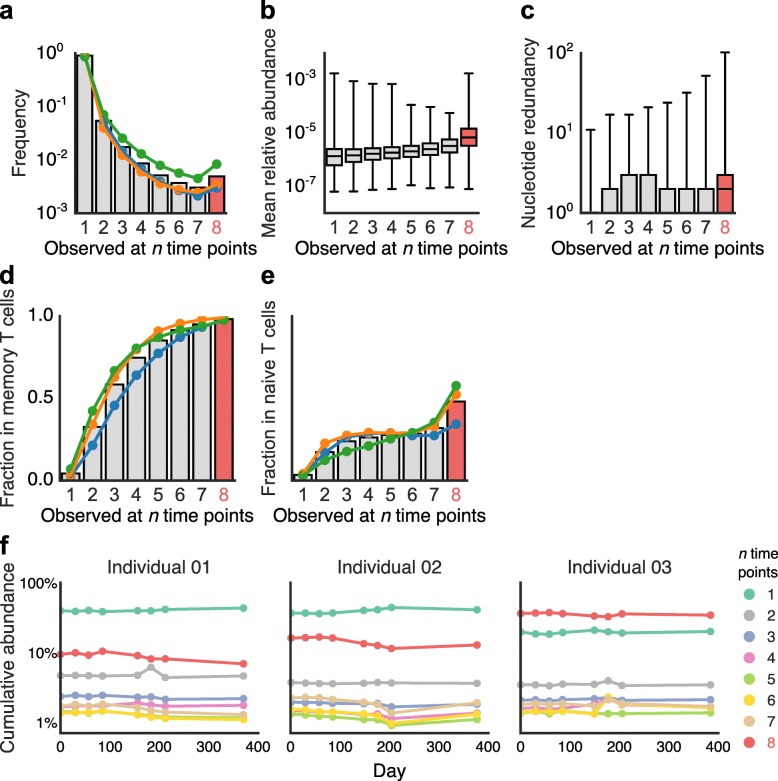
A subset of the TCRβ repertoire occurred across all time points—the persistent TCRβ repertoire. **a** The number of TCRβs observed at *n* time points. Persistent TCRβs tended to have (**b**) greater abundance (Mann-Whitney *U* test, statistic = 26,297,052,589.5, *p* < 10^− 308^) and (**c**) nucleotide sequence redundancy (Mann-Whitney *U* test, statistic = 25,851,211,348.0, *p* < 10^− 308^) than other receptors. Mann-Whitney *U* tests between groups are in Additional file 2: Tables S6, S7. Persistent TCRβs had higher proportions of TCRβs in common with memory (**d**) and with naive (**e**) T cell populations and constituted a stable and significant fraction of overall TCRβ abundance across time (**f**)

During our analysis, we discovered a subset of TCRβs that was present across all eight PBMC samples from a single individual, a subset we called “persistent” TCRβs (Fig. 2a). While approximately 90% of unique TCRβs observed over all of an individual’s PBMC samples occurred in only one sample, 0.3–0.8% of TCRβs occurred at all eight time points (Fig. 2a). When considering individual samples, this pattern translated to 1–5% of TCRβs observed in each sample were persistent receptors (Additional file 2: Table S5). When we considered only high-abundance TCRβs, the frequency of persistent TCRβ increased substantially (Additional file 1: Figure S8a).

We hypothesized that these persistent TCRβs might be selected for and maintained by the immune system, perhaps to respond to continual antigen exposures or other chronic immunological needs.

In our data, we found multiple signatures of immunological selection acting on persistent TCRβs. The members of this persistent subset tended to have a higher mean abundance than TCRβs observed at fewer time points (Fig. 2b, Additional file 2: Table S6). We also observed that the number of unique nucleotide sequences encoding each TCRβ’s CDR3 amino acid sequence was generally higher for persistent TCRβs (Fig. 2c, Additional file 2: Table S7). This pattern of greater nucleotide redundancy varied across individuals and region of the CDR3 sequence (Additional file 1: Figure S9a), but TCRβs with the highest nucleotide redundancy were reliably persistent (Additional file 1: Figure S9b). Furthermore, we discovered that TCRβs occurring at more time points, including persistent TCRβs, shared larger proportions of TCRβs also associated with memory T cells (Fig. 2d). Remarkably, 98% of persistent TCRβs also occurred in memory T cells, suggesting that almost all persistent T cell clones had previously encountered and responded to their corresponding antigens. We found a similar pattern in naive T cells, although the overall overlap was lower (50%), indicating that persistent TCRβs were also enriched in the naive compartment (Fig. 2e). Persistent TCRβs did not show altered CDR3 lengths or VJ usage (Additional file 1: Figures S10-S12). Like alpha diversity and clonality, the cumulative abundance of TCRβs present in different numbers of samples appeared stable over time and specific to individuals (Fig. 2f). Surprisingly, although persistent TCRβs constituted less than 1% of all unique TCRβs, they accounted for 10–35% of the total abundance of TCRβs in any given sample (Fig. 2f), further evidence that these T cell clones had expanded. We observed similar patterns when analyzing only high-abundance TCRβs (Additional file 1: Figure S8).

Taken together, these characteristics—persistence across time, higher abundance, redundant nucleotide sequences, and overlap with memory T cells—suggest immunological selection for persistent TCRβs. We therefore investigated whether persistent TCRβs coexisted with TCRβs having very similar amino acid sequences. Previous studies have suggested that TCRβs with similar sequences likely respond to the same or similar antigens, and such coexistence may be evidence of immunological selection [25, 26].

**Fig. 3:**
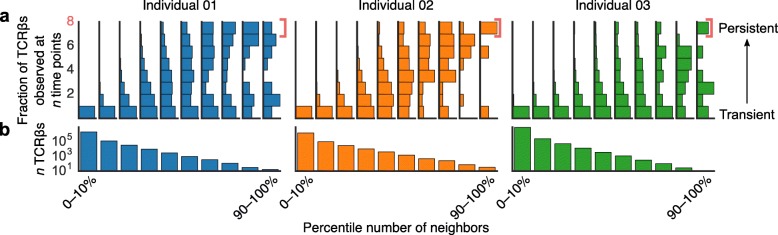
Persistent TCRβs were more functionally redundant. We created a network graph of TCRβs from each individual, drawing edges between TCRβs on the basis of sequence similarity (Levenshtein distances), which reflects antigen specificity. We then grouped TCRβs into decile bins based on the number of neighbors (similar TCRβs) of each TCRβ. In other words, TCRβs in the 0–10% bin had 0 to 10% of the maximum number of neighbors observed for any TCRβ—the fewest neighbors—while those in the 90–100% bin had near the maximum number of neighbors observed. For each decile bin, we then counted how many samples each TCRβ occurred in from our time series data. **a** Vertical histograms of these distributions indicate that TCRβs with few neighbors —and thus few similar observed TCRβs—tended to occur at only a single time point, while TCRβs with more neighbors—and thus higher numbers of similar TCRβs observed—tended to have a higher proportion of persistent TCRβs. **b** The number of TCRβs in each neighbor bin (Additional file 1: Figure S13a)

To explore this idea, we applied a network clustering algorithm based on Levenshtein edit distance between TCRβ CDR3 amino acid sequences in our data [25–27]. We represented antigen-specificity as a network graph of unique TCRβs, in which each edge connected a pair of TCRβs with putative shared specificity. We found that TCRβs having few edges—and thus few other TCRβs with putative shared antigen specificity—tended to occur in only one sample, while TCRβs with more edges included a higher frequency of TCRβs occurring in more than one sample (Fig. 3a, *p* < 10^− 5^ for all three individuals by a nonparametric permutation test). This pattern indicates that TCRβs occurring with other, similar TCRβs were more often maintained across time in the peripheral immune system.

**Fig. 4:**
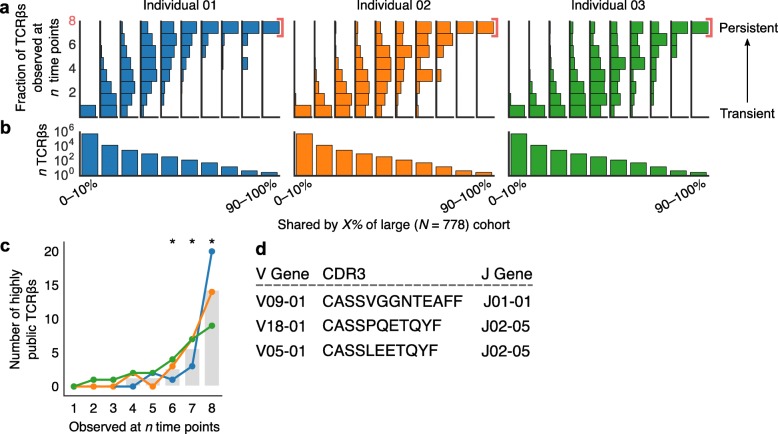
Persistent TCRβs were enriched in highly public TCRβs. We identified public TCRβs occurring in 0–10%, 0–20%, . . . 90–100% of individuals in an independent, large cohort of similarly profiled subjects (*N* = 778). For each of these decile bins, we examined TCRβs shared across each of our three individuals’ time series data and tallied the number of time points at which we observed each TCRβ. **a** Vertical histograms of these distributions indicate that more-private TCRβs—TCRβs shared by few people—occurred most often at only a single time point, while more-public TCRβs tended to persist across time. **b** The number of TCRβs evaluated in each decile bin. The vast majority of receptors were not shared or were shared across few individuals (also see Additional file 1: Figure S13b). **c** In all three individuals in this study, persistent TCRβs included greater numbers of highly public TCRβs—defined here as receptors shared by over 70% of subjects from the large cohort—than receptors that only occurred once (independent t-test, statistic = − 4.508, *p* = 0.01). Asterisks indicate *p* < 0.05. **d** The three most public TCRβs (in over 90% of 778 individuals) were also persistent in all three individuals

We next examined the association between persistent TCRβs—those shared across time points—and “public” TCRβs—those shared across people. Public TCRs show many of the same signatures of immunological selection as persistent TCRβs, including higher abundance [28], overlap with memory T cells [28], and coexistence with TCRs with similar sequence similarity [25]. To identify public TCRβs, we compared our data with a similarly generated TCRβ dataset from a large cohort of 778 healthy individuals [21] (Additional file 4). We found that the most-shared (i.e., most-public) TCRβs from this large cohort had a larger proportion of persistent TCRβs from our three sampled individuals (Fig. 4a–b, Additional file 2: Table S8, *p* < 10^− 5^ for all three individuals by a nonparametric permutation test). Private TCRβs—those occurring in few individuals—most often occurred at only a single time point in our analyses. Interestingly, TCRβs that occurred at many but not all time points (i.e., 3–5 time points) were on average the most-shared (Additional file 1: Figure S14a), but persistent TCRβs were specifically enriched in highly public TCRβs—here defined as those shared by over 70% of subjects in the large cohort (Fig. 4c, Additional file 1: Figure S14b). The three most public TCRβs (found in over 90% of the 778-individual cohort) were found to be in the persistent TCRβ repertoires of all three individuals and were diverse in structure (Fig. 4d).

Public TCRs are thought to be products of genetic and biochemical biases in T cell receptor recombination [29–31] and also of convergent selection for TCRs that respond to frequently encountered antigens [21, 32]. To better understand the effects of biases during TCRβ recombination on receptor persistence, we used IGoR to estimate the probability that each TCRβ was generated before immune selection [33]. Similar to previous studies [30], the probability that a given TCRβ was generated correlated closely with publicness (Additional file 1: Figure S15a). In our time series data, TCRβs that occurred at multiple time points tended to have slightly higher generation probabilities than TCRβs only observed once (Additional file 1: Figure S15b), but persistent TCRβs did not have higher generation probabilities than other receptors observed in more than one time point. In addition, more abundant TCRβs (both persistent and nonpersistent) did not have higher generation probabilities (Additional file 1: Figure S15c–d). These results suggest that, unlike public receptors, persistent receptors and their abundances do not appear to result from biases in TCR recombination. The contradiction that public and persistent receptors are associated but only public TCRβs appear to be generated by recombination bias is possible because despite their association, these two TCRβ subsets are largely independent. Although the most public receptors are overwhelmingly persistent (Fig. 4), they represent a tiny fraction of the persistent receptors in each individual. Thus, although these two subsets of the TCR repertoire—persistent and public—overlap and share many characteristics, they are also distinct, suggesting that they may play complementary roles in adaptive immunity.

## Discussion

Our analyses revealed both fluctuation and stability in the TCRβ repertoire of healthy individuals, providing a baseline framework for interpreting changes in the TCR repertoire. We identified a number of consistent repertoire characteristics (e.g., diversity, clonality), which are known to be affected by immunizations, clinical interventions, and changes in health status [7, 14, 34]. These patterns differed among individuals across time, highlighting the role played by genetics [like human leukocyte antigen (HLA) type] and history of antigen exposure in shaping the TCR repertoire. We did not obtain HLA-type information from these three subjects, so the relative contributions of HLA type versus individual history remains unknown.

We further discovered a subset of persistent TCRβs that bore signs of immune selection. Persistent TCRβs tended to be more abundant than nonpersistent receptors, although this distinction is to a certain extent confounded by the fact that high-abundance receptors are also more likely to be detected in a given sample. Nevertheless, this circular logic does not detract from the immune system’s maintenance of specific dominant TCRβs across time. We further found that persistent TCRβs had higher numbers of distinct nucleotide sequences encoding each TCRβ. TCR diversity is generated by somatic DNA recombination, so it is possible for the same TCR amino acid sequence to be generated from independent recombinations in different T cell clonal lineages. Thus, coexistence of multiple clonal lineages encoding the same TCRβ amino acid sequence may reflect selective pressures to maintain that TCRβ and its antigen specificity. Similarly, the presence of many TCRβs similar to persistent TCRβs—as identified by our network analysis—could also result from selection for receptors that recognize a set of related antigens [20, 35]. Previous studies using network analyses also found that public TCRβs tend to occur with similar TCRβs [25], further suggesting that both public and persistent TCRβs are key drivers of lasting immunity. In addition to using TCRβ sequencing to track TCRβs that proliferate in response to intervention, we propose that the three dimensions explored in this paper—similarity with other receptors, publicness across individuals, and persistence through time—represent useful strategies for identifying biologically important TCRβs.

The presence of near-ubiquitous (present in > 90% of individuals in a cohort of 778 individuals) and persistent TCRβs led us to speculate that these TCRβs might be responding to a set of common antigens repeatedly encountered by healthy people. These antigens could be associated with self-antigens, chronic infections (e.g., Epstein-Barr virus), or possibly members of the human microbiota. In fact, the CDR3 sequence CASSPQETQYF has been previously been associated with the inflammatory skin disease psoriasis [36] and CASSLEETQYF has been implicated in responses to *Mycobacterium tuberculosis* [20] and cytomegalovirus [37].

In addition to persistent TCRβs, our analysis revealed many receptors with unstable, transient behavior. Many high-abundance TCRβs did not persist through time, with many occurring at only a single time point (Fig. 2b, Additional file 1: Figure S8a). These TCRβs could well correspond to T cells that expanded during a temporary immune challenge but then did not persist in high abundance afterward. These dynamics might also reflect the migration of T cells to and from different tissues, which could manifest as fluctuating abundance in the blood. The presence of dynamically expanding or migrating TCRβs in apparently healthy individuals poses an important consideration for designing studies monitoring the immune system. Studies tracking TCR abundances in cross-sectional immune system sampling [7, 14, 34, 35, 38–41] may capture not only T cell clones responding to intervention, but also expanding clones inherent in the T cell dynamics of healthy individuals. Repeated sampling before and after intervention could minimize such false positives.

Current immunosequencing methods have limitations that should inform the interpretation of our results. Most important, given such a diverse system as the TCR repertoire, even large sequencing efforts like ours undersample. Although our sequencing appeared to saturate our samples (Additional file 1: Figure S2), additional bottlenecks during library preparation and, particularly, blood drawing limit our ability to capture full TCRβ diversity. Previous studies exhaustively sequenced multiple libraries from multiple blood samples, but even these estimates are considered a lower limit of TCRβ diversity [42]. This detection limit could confound our identification of persistent TCRβs. Many of the TCRβs that did not occur in all samples were undoubtedly present but too rare for our analysis to capture. Thus, identification of a persistent TCR repertoire was subject to an abundance cutoff, whereby we focused on TCRs that persisted above the detection limit of sampling. To check that our conclusions were not heavily altered by undersampling, we analyzed high-abundance TCRβs and found similar overall patterns, so we infer that our main conclusions are likely robust despite this experimental limitation. In addition, our study included data from only three female individuals ages 18–45. The immune system varies across sex [43] and age [44], and although the patterns we describe are clear, larger longitudinal studies on the immune repertoire with greater patient characterization (particularly HLA type) and representation (e.g., including men and a range of ages) will better define how these patterns apply across populations.

## Conclusions

To better understand healthy immune system dynamics in humans, we profiled the TCRβ repertoires from three individuals over one year. We found a system characterized by both fluctuation and stability and further discovered a novel subset of the TCRβ repertoire that might play a key role in immunity. As immune profiling in clinical trials becomes more prevalent, we hope our results will provide much-needed context for interpreting immunosequencing data, as well as for informing future trial designs.

## Methods

### Study design

We sought to study baseline dynamics and characteristics of the TCRβ repertoire in healthy individuals across time. We sampled blood from three individuals from eight time points over one year. We kept our sample size small so that we could perform extremely deep immune repertoire profiling on each sample, a choice that should be taken into consideration when interpreting our results.

### Sample collection

Three healthy adult female volunteers ages 18–45 provided blood samples over of one year, with samples taken on a starting date and 1, 2, 3, 5, 6, 7, and 12 months after that date (Fig. 1a). We sequenced TCRβ chains from approximately 1 million PBMCs from each sample. From the samples at 5, 6, and 7 months, we also sequenced TCRβ chains from sorted naive (CD3+, CD45RA+) and memory (CD3+, CD45RO+) T cells.

### High-throughput TCRβ sequencing

We extracted genomic DNA from cell samples using a Qiagen DNeasy blood extraction kit (Qiagen, Gaithersburg, MD, USA). We sequenced CDR3 regions of rearranged TCRβ genes and defined these regions according to the international immunogenetics information system (IMGT) [45]. We amplified and sequenced TCRβ CDR3 regions using previously described protocols [2, 46]. Briefly, we applied a multiplexed PCR method, using a mixture of 60 forward primers specific to TCR Vβ gene segments plus 13 reverse primers specific to TCR Jβ gene segments. We sequenced 87 base-pair reads on an Illumina HiSeq System and processed raw sequence data to remove errors in the primary sequence of each read. To collapse the TCRβ data into unique sequences, we used a nearest-neighbor algorithm—merging closely related sequences—which removed PCR and sequencing errors. By sequencing genomic DNA and not RNA, our approach more accurately reflected T cell abundances but also captured both expressed and unexpressed T cell receptors [19].

### Data analysis

In our analyses, we focused on TCRβs containing no stop codons and mapping successfully to a V gene and J gene (Additional file 2: Table S1). Relative abundances of these “productive” TCRβ sequences, however, took into account the abundances of nonproductive TCRβ sequences, as these sequences were still part of the greater TCRβ pool. We defined a TCRβ as a unique combination of V gene, J gene, and CDR3 amino acid sequence. We examined nucleotide redundancy of each TCRβ by counting the number of T cell clones—a unique combination of V gene, J gene, and CDR3 nucleotide sequence—encoding each TCRβ. We defined TCRβs whose abundances ranked in the top 1% for each sample as high-abundance TCRβs, and we analyzed these TCRβs in parallel with the full TCRβ repertoire as a check for artifacts of undersampling (Additional file 1: Figures S5, S8).

We calculated Spearman’s and Pearson’s correlation coefficients for TCRβ abundances across samples using the Python package SciPy, considering only TCRβs that were shared among samples. We calculated alpha diversity (Shannon estimate = *e*^(Shannon entropy)^) and clonality (1 – Pielou’s evenness) using the Python package Scikit-bio 0.5.1. We calculated Levenshtein distance using the Python package Python-Levenshtein 0.12.0 and analyzed the resulting network using the Python package NetworkX 1.9.1.

To look for TCRβs with similar temporal dynamics, we focused on TCRβs that occurred in the top 1% at least twice. These TCRβs likely represented T cell clones that had expanded. We then calculated Spearman’s and Pearson’s correlation coefficients for all high-abundance TCRβ pairs, filling in missing data with the median abundance of TCRβs from each sample. We used median abundance—instead of a pseudocount of 1 or half the minimum abundance detected—because the immense diversity of the TCRβ repertoire means that most detected TCRβs are likely similarly abundant as TCRβs that were not detected. We identified pairs of TCRβs that had high (> 0.95) correlation. To identify cohorts of TCRβs that co-correlated, we represented TCRβs as nodes in a network, where nodes were connected by edges if the corresponding TCRβs were highly correlated. We then searched for the maximal network clique (a set of nodes where each node has an edge to all other nodes) using NetworkX. We visually inspected these TCRβ cohorts for evidence of sequencing error, which might have resulted in a high-abundance TCRβ that closely correlated with many low-abundance TCRβs with similar sequences (Additional file 2: Table S4). To test the significance of TCRβ cohort size, we performed the same analysis on 1000 shuffled datasets. Each shuffled dataset randomly permuted sample labels (i.e., the sampling date) for each TCRβ within each individual.

To test the significance of persistent TCRβ enrichment in (a) public receptors (Fig. 4) and (b) TCRβs that occurred with many similar receptors (Fig. 3), we analyzed 10,000 shuffled datasets. For these permutations, we randomly permuted the number of time points at which each TCRβ was observed and repeated the analysis.

We estimated the probability of generation of each TCRβ before to immune selection using IGoR version 1.1.0 with the provided model parameters for the human TCRβ locus [33].

## Additional files

Additional file 1: **Figure S1.** Representative frequency rank plots for memory T cells, naive T cells, and all T cells from PBMCs from Individual 01. **Figure S2.** Rarefaction curves for each subject indicate that sample libraries were sequenced well past saturation. **Figure S3.** Analyses examining only high-abundance TCRβs agree with results from full-repertoire analysis, suggesting that undersampling likely did not confound our results. **Figure S4.** TCRβ repertoire overlap (Jaccard index) often decreases with increasing time between samples. **Figure S5.** V gene usage across time and cell compartment in all three individuals. **Figure S6.** J gene usage across time and cell compartment in all three individuals. **Figure S7.** Cohorts of TCRβs exhibit correlated dynamics over time. **Figure S8.** Persistent high-abundance TCRβs exhibit similar patterns as overall persistent TCRβs. **Figure S9.** Nucleotide redundancy across individuals and with more stringent assignment of CDR3 sequence. **Figure S10.** The persistent TCRβ repertoire exhibited little alteration of CDR3 lengths. **Figure S11.** The persistent TCRβ repertoire does not exhibit altered V gene usage. **Figure S12.** The persistent TCRβ repertoire does not exhibit altered J gene usage. **Figure S13.** Distributions of the number of neighbors and degree of sharing across people for all TCRβs and high-abundance TCRβs. **Figure S14.** Persistent TCRβs were rich in highly public TCRβs. **Figure S15.** Persistent and public receptors may result in part from TCR recombination biases. (DOCX 3566 kb)

Additional file 2: **Table S1.** Overall TCRβ-sequencing statistics per sample: sequencing depth, productive TCRβ sequencing depth, fraction of productive TCRβ sequences, unique V genes identified, unique J genes identified, unique CDR3 sequences, unique TCRβs, unique TCRβ nucleotide sequences. **Table S2.** V gene usage across subject and T cell population, expressed as both a fraction of all unique productive TCRβs and as a mean total abundance per sample. **Table S3.** J gene usage across subject and T cell population, expressed as both a fraction of all unique productive TCRβs and as a mean total abundance per sample. **Table S4.** Sequence and abundance information for the largest cohort of closely correlated TCRβs identified in each individual by Spearman’s or Pearson’s correlation. **Table S5.** The fraction of TCRβs in each sample that occurred in 1–8 samples from that subject’s time series. **Table S6.** Mann-Whitney *U* test statistics for mean abundance of TCRβs occurring in different numbers of samples during the time series. **Table S7.** Mann-Whitney *U* test statistics for nucleotide redundancy of TCRβs occurring in different numbers of samples during the time series. **Table S8.** The fraction of TCRβs in each sample that were shared to different degrees among subjects in a large, independent cohort. (XLSX 80 kb)

Additional file 3: Count, frequency, V, J, and CDR3 amino acid sequence data for high abundance TCRβs in each sample. This is a tab-delimited, gzip-compressed file. The column “tcr” corresponds to a label that identifies a unique combination of CDR3 amino acid sequence, V gene, and J gene observed in this study. The column “count (templates/reads)” represents the counts of a given receptor’s DNA sequence in the sequencing data. The column “frequencyCount (%)” is the relative abundance of that tcr within each sample, accounting for productive and nonproductive receptors. The columns “vGeneName” and “jGeneName are the IMGT assigned V and J genes. The column “aminoAcid” is the CDR3 amino acid sequence. (TSV 8820 kb)

Additional file 4: Data for persistence across our time series and sharing across subjects in an independent, large cohort for TCRβs in this study. The columns “aminoAcid”, ‘vGeneName”, “jGeneName”, and “tcr” are the same as for Additional file 3. The column “n_cmv_public” is the number of subjects (out of 778) that shared that TCRβ. The columns “num_occ_sub1_pbmc”, “num_occ_sub2_pbmc”, and “num_occ_sub3_pbmc” are the number of time points at which a given receptor was observed in the PBMC samples for Individual 01, 02, and 03, respectively. (TSV 60870 kb)

## Data Availability

The dataset supporting the conclusions of this article is available in the immuneACCESS portal of Adaptive Biotechnologies repository, https://clients.adaptivebiotech.com/pub/healthy-adult-time-course-TCRB.

## Abbreviations

D: Diversity gene or region of the T cell receptor
HLA: Human leukocyte antigen
J: Joining gene or region of the T cell receptor
PBMC: Peripheral-blood mononuclear cell
TCR: T cell receptor
TCRβ: T cell receptor beta chain
V: Variable gene or region of the T cell receptor
