# Supplementary material for: Longitudinal immunosequencing in healthy people reveals persistent T cell receptors rich in highly public receptors
Source: BMC Immunol. 2019 Jun 21;20:19. doi: 10.1186/s12865-019-0300-5 (PMC6588944; doi:10.1186/s12865-019-0300-5)
Supplement: Supplementary file 1 — Figure S1. Representative frequency rank plots for memory T cells, naive T cells, and all T cells from PBMCs from Individual 01. Figure S2. Rarefaction curves for each subject indicate that sample libraries were sequenced well past saturation. Figure S3. Analyses examining only high-abundance TCRβs agree with results from full-repertoire analysis, suggesting that undersampling likely did not confound our results. Figure S4. TCRβ repertoire overlap (Jaccard index) often decreases with increasing time between samples. Figure S5. V gene usage across time and cell compartment in all three individuals. Figure S6. J gene usage across time and cell compartment in all three individuals. Figure S7. Cohorts of TCRβs exhibit correlated dynamics over time. Figure S8. Persistent high-abundance TCRβs exhibit similar patterns as overall persistent TCRβs. Figure S9. Nucleotide redundancy across individuals and with more stringent assignment of CDR3 sequence. Figure S10. The persistent TCRβ repertoire exhibited little alteration of CDR3 lengths. Figure S11. The persistent TCRβ repertoire does not exhibit altered V gene usage. Figure S12. The persistent TCRβ repertoire does not exhibit altered J gene usage. Figure S13. Distributions of the number of neighbors and degree of sharing across people for all TCRβs and high-abundance TCRβs. Figure S14. Persistent TCRβs were rich in highly public TCRβs. Figure S15. Persistent and public receptors may result in part from TCR recombination biases. (DOCX 3566 kb) [file 12865_2019_300_MOESM1_ESM.docx]

**Longitudinal immunosequencing in healthy people reveals persistent T cell receptors rich in highly public receptors**

**Authors:** Nathaniel D. Chu, Haixin Sarah Bi, Ryan O. Emerson, Anna M. Sherwood, Michael E. Birnbaum, Harlan S. Robins, Eric J. Alm

**Supporting Information**


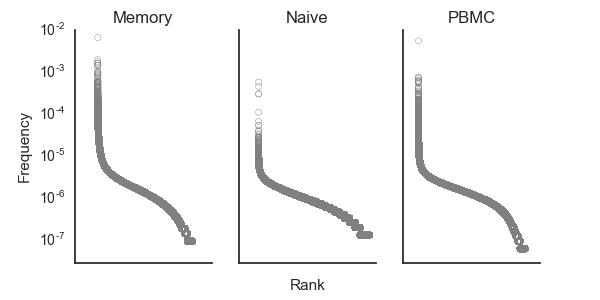


**Figure S1**. Representative frequency rank plots for memory T cells, naive T cells, and all T cells from PBMCs from Individual 01. As expected, naive T cells had fewer abundant clones than PBMC or memory T cells. In all cases, the majority of TCRβs had abundances around 10^–6^.


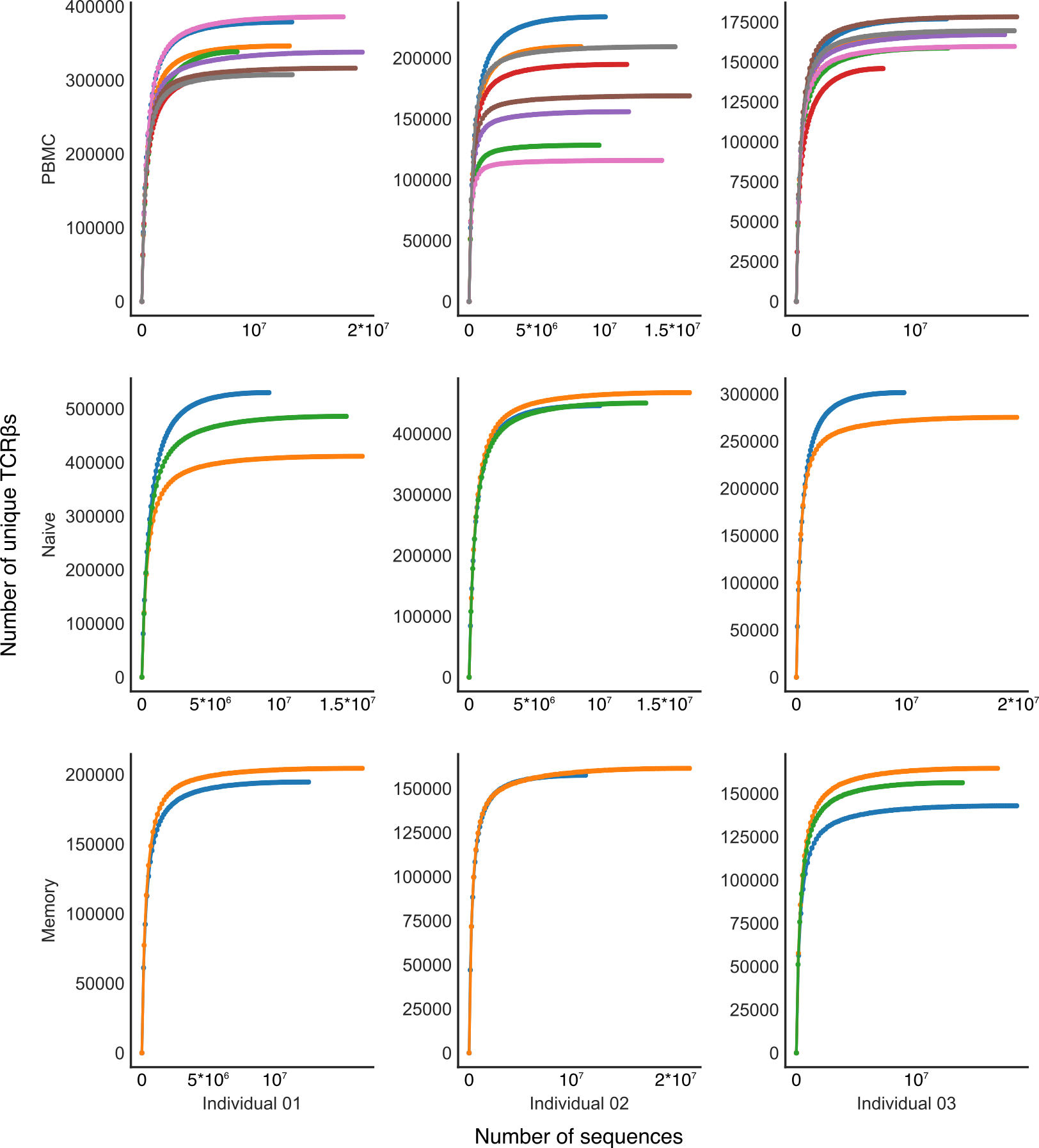


**Figure S2**. Rarefaction curves for each subject indicate that sample libraries were sequenced well past saturation.


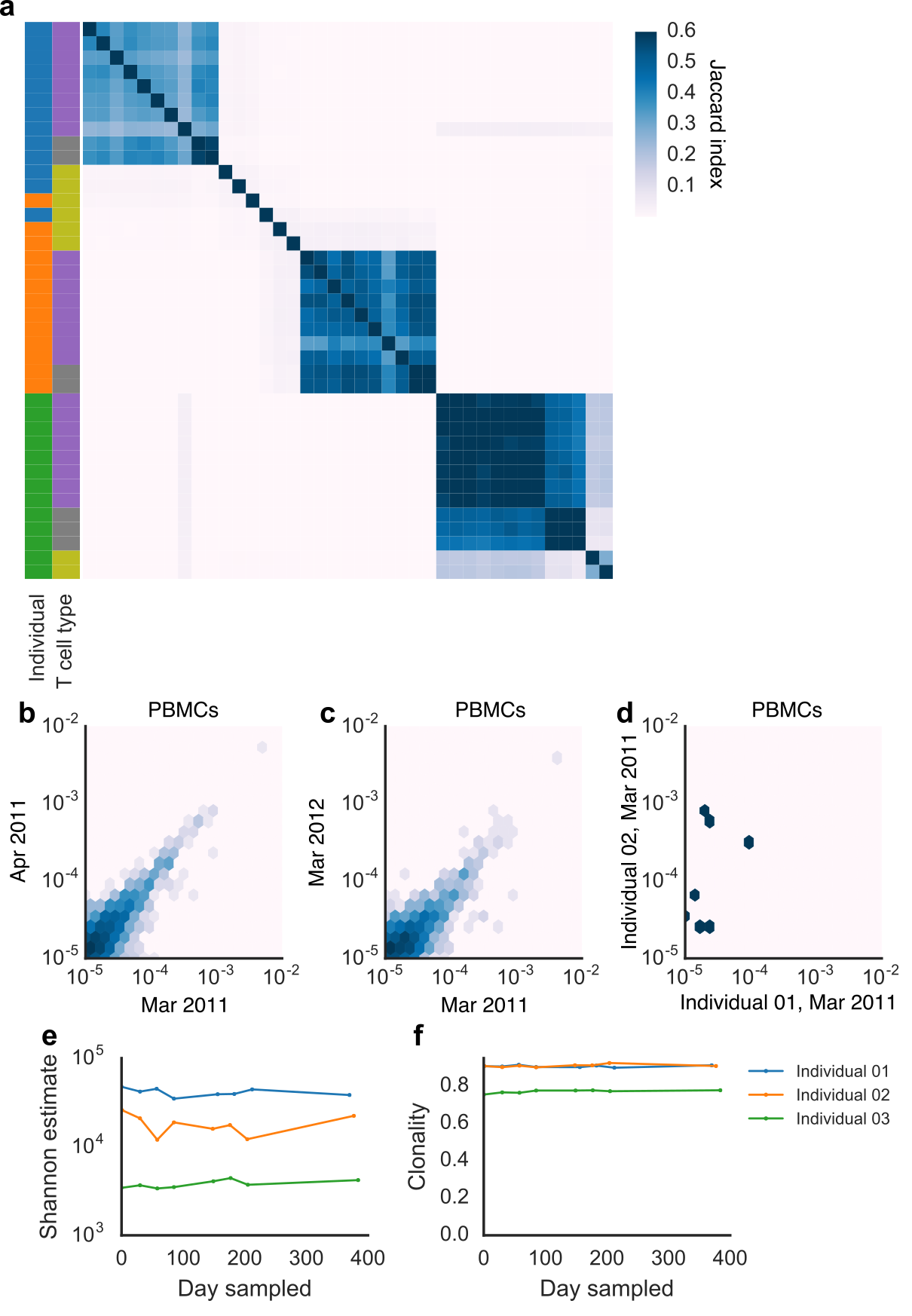


**Figure S3**. Analyses examining only high-abundance TCRβs agree with results from full-repertoire analysis, suggesting that undersampling likely did not confound our results. (**a**) A heatmap of Jaccard indexes shows similar clustering of PBMC and memory T cell samples by individual and less clustering of naive T cell samples. Abundances of high-abundance TCRβs in PBMC samples correlated within an individual (individual 01) across time points, including across a month (**b**, shared TCRβs = 2057, Spearman *rho* = 0.66902, *p* < 10^–6^) and a year (**c**, shared TCRβs = 1390, Spearman *rho* = 0.59251, *p* < 10^–6^). High-abundance TCRβs did not appear to correlate across individuals, largely because of lack of shared TCRβs (**d**, shared TCRβs = 7, Spearman *rho* = 0.14286, *p* = 0.75995). Shannon alpha diversity estimate (**e**) and clonality (defined as 1 – Pielou’s evenness, **f**) of the TCRβ repertoire were consistent over time.


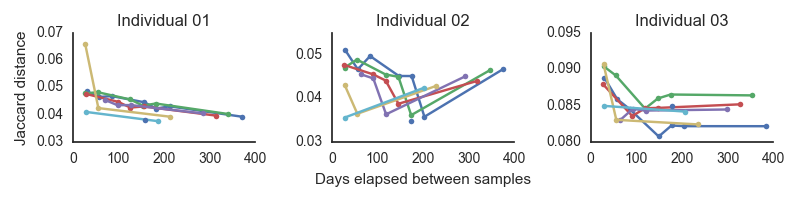


**Figure S4**. TCRβ repertoire overlap (Jaccard index) often decreases with increasing time between samples, except in Individual 02, where the final time point at one year past the first sample shared more TCRβs with the previous samples. Different colors depict changes over time relative to each sample. For example, the blue line depicts overlap between each sample after the first sample and the first sample, while the green line depicts overlap between each sample after the second sample and the second sample


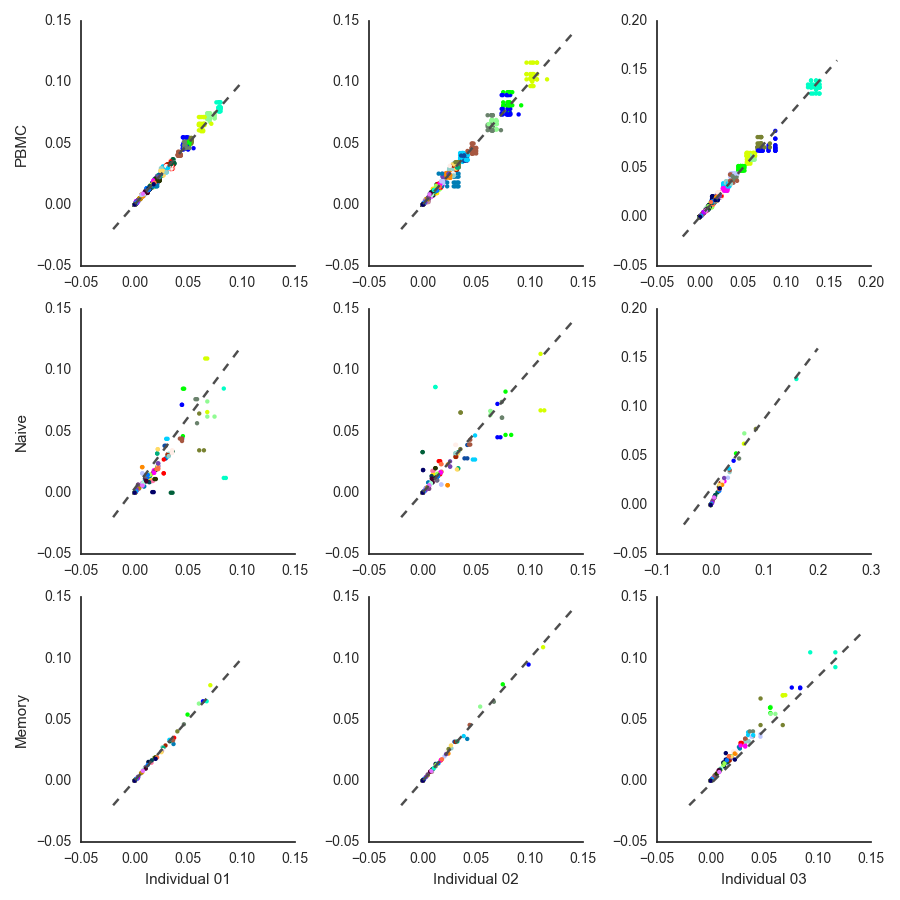


**Figure S5**. V gene usage across time and cell compartment in all three individuals. Each of nine plots represents each cell type in each individual. Within each plot, different colors represent different V genes, and each dot represents a comparison of the abundances of that V gene from one sample to another. Points that fall near a 1:1 ratio (indicated by the dotted line) are nearly identical in abundance between the two samples considered. These plots indicate that VJ gene usage was generally the same across time points, particularly in total and memory T cells. In naive cells, VJ gene usage varied more.


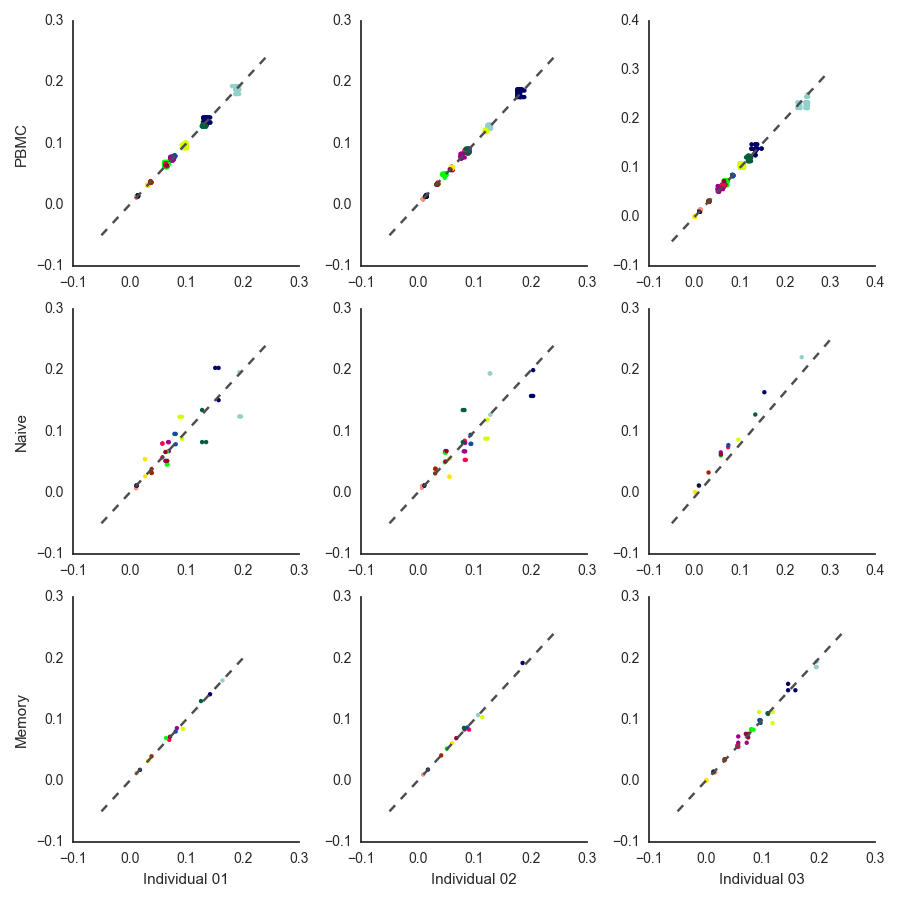


**Figure S6**. J gene usage across time and cell compartment in all three individuals. Plots are as in **Figure S5**, with similar findings.


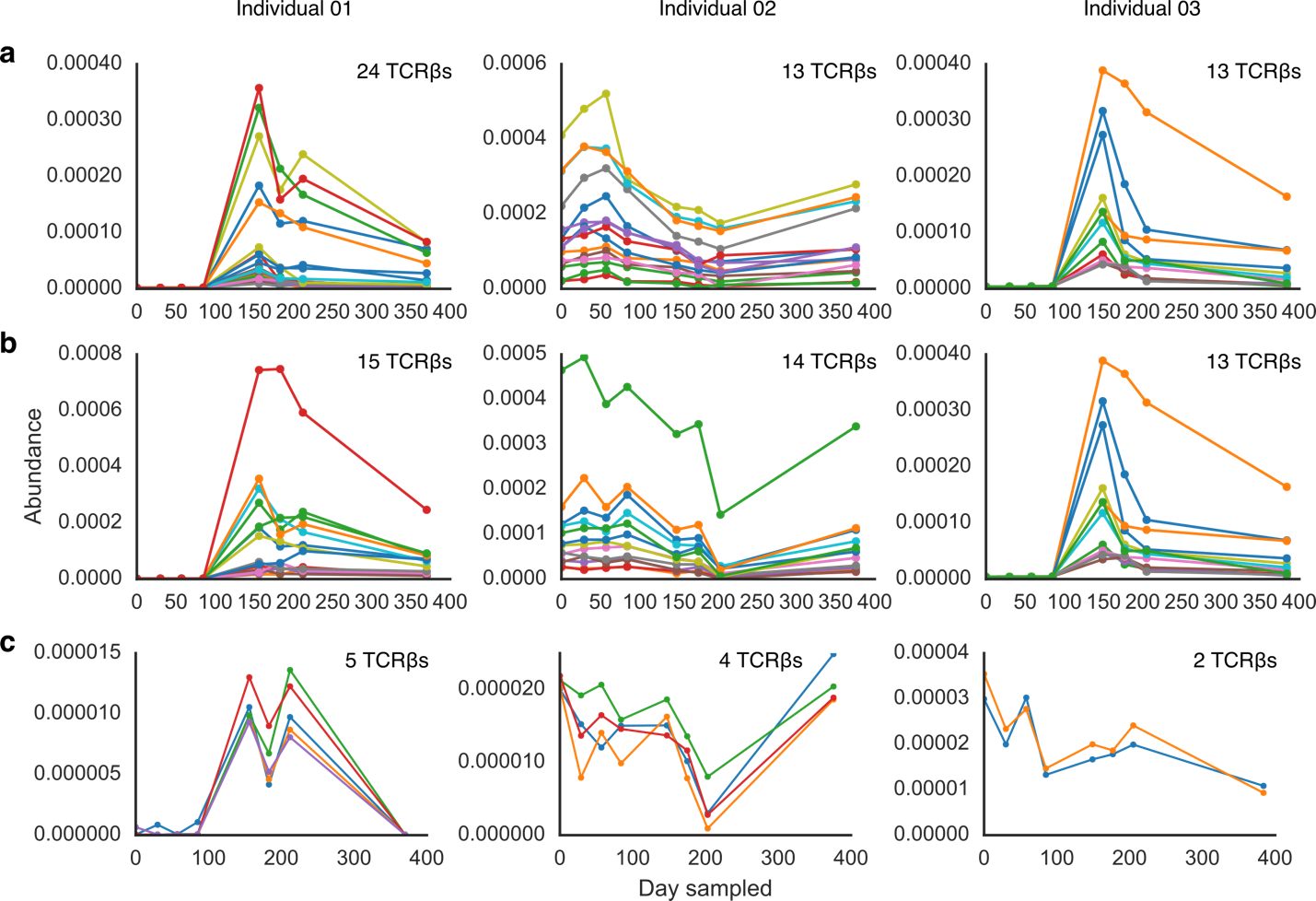


**Figure S7**. Cohorts of TCRβs exhibit correlated dynamics over time. We found large cohorts of correlating TCRβs by Spearman (**a**) and Pearson (**b**) correlation. Although these TCRβs spanned a range of abundances, we did not observe any clear signs of correlation caused by sequencing or library preparation errors (**Table S2**). We also found smaller cohorts (**c**) of TCRβs with nearly identical abundances whose dynamics also correlated through time. The number of TCRβs found in all cohorts was significant (*p* < 0.001) in a random permutation test (see Methods). These TCRβ cohorts might be an artifact of sampling noise, or they may represent receptors involved in the same immune response.


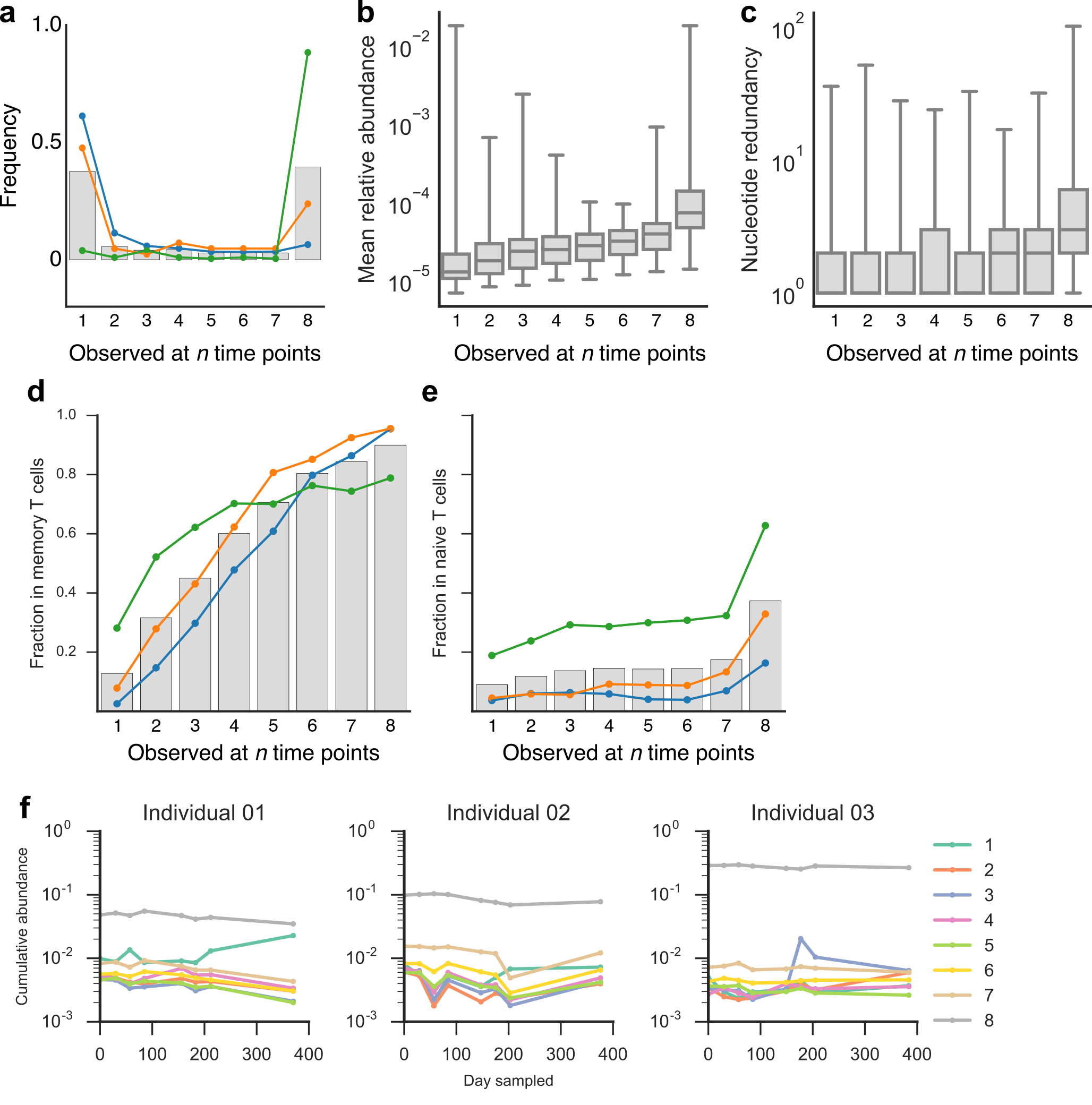


**Figure S8**. Persistent high-abundance TCRβs exhibit similar patterns as overall persistent TCRβs. (**a**) High-abundance TCRβs had a greater prevalence of persistent TCRβs, although the exact values varied across individuals. Persistent high-abundance TCRβs also showed greater mean abundance (**b**) and nucleotide redundancy (**c**). Persistent high-abundance TCRβs also had higher proportions of TCRβs in common with memory (**d**) and naive (**e**) T cell populations and constituted a stable and significant fraction of overall TCRβ abundance across time (**f**).


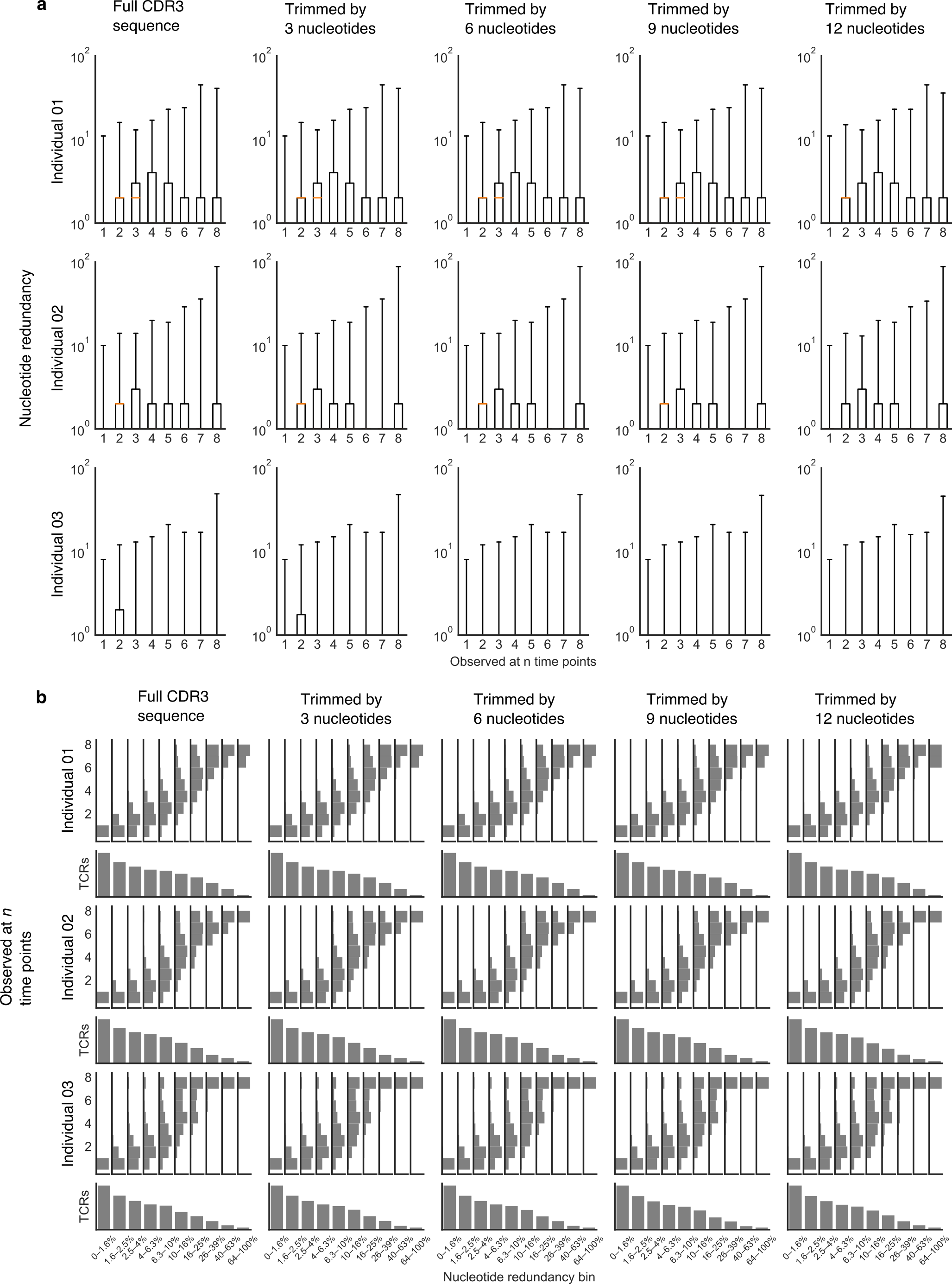


**Figure S9**. Nucleotide redundancy across individuals and with more stringent assignment of CDR3 sequence (figure supplements Figure 2c). (**a**) Each plot represents nucleotide redundancy for TCRβs that were observed in *n* samples. Rows represent plots for each individual. The left-most column of plots comprises data from full CDR3 nucleotide sequences as identified by IMGT (as in Figure 2c): we observed that the pattern of increasing nucleotide redundancy in persistent TCRβs was not consistent across individuals. Each of the following columns plot data from CDR3 nucleotide sequences that were progressively trimmed on each end by 3, 6, 9, and 12 nucleotides. We trimmed these sequences because CDR3 sequences identified by IMGT generally capture a number of amino acids—usually one to four at each end of the sequence—that are derived from V and J genes. Nucleotide mutations in these leading and trailing ends are thus less likely to be of biological origin and more likely to be from sequencing error, since we do not expect nucleotides from the V or J genes to be altered during TCR recombination (except for deletions). From these plots, we can observe that nucleotide redundancy is generally stable over different lengths of trimming, suggesting that our data are not skewed by these potential sequencing errors. (**b**) To further examine the relationship between persistence and nucleotide redundancy, we grouped TCRβs into 10 bins according to nucleotide redundancy. Because nucleotide redundancy is extremely skewed—the vast majority of TCRβs are encoded by a single clonotype—we created these bins on a logarithmic scale: the first bin includes TCRβs with nucleotide redundancy values up to 1.6% of the maximum value for each individual; the second between 1.6% and 2.5% of the maximum value; and up to the 10th bin, which includes TCRβs with nucleotide redundancy values between 64% and 100% of the maximum value. For each of these TCRβ bins, we then plotted a histogram of the frequency of TCRβs that were observed at *n* time points. We observe a clear pattern across individuals and trimming lengths: TCRβs with greater nucleotide redundancy tend to occur at more time points, and the most redundant TCRβs are exclusively persistent receptors.


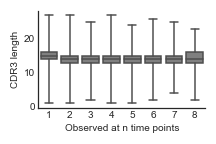


**Figure S10**. The persistent TCRβ repertoire exhibited little alteration of CDR3 lengths.


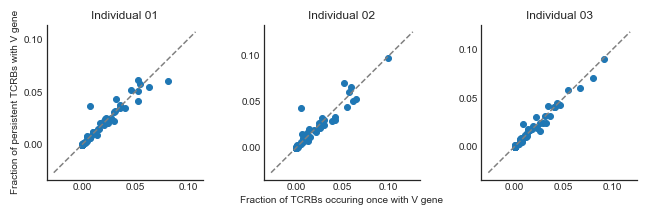


**Figure S11**. The persistent TCRβ repertoire does not exhibit altered V gene usage. These plots show V gene usage in TCRβs that occurred only once (x-axis) versus in persistent TCRβs (y axis). Each data point represents a single V gene. These values were closely correlated.


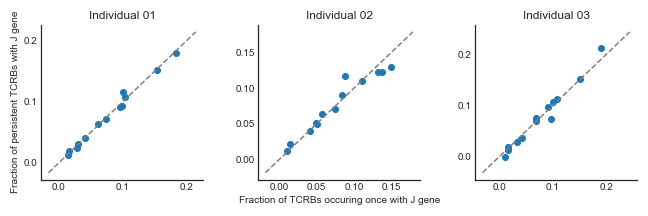


**Figure S12**. The persistent TCRβ repertoire does not exhibit altered J gene usage. Similar plots as in **Figure S10** indicate that J gene usage is not greatly changed in persistent TCRβs.


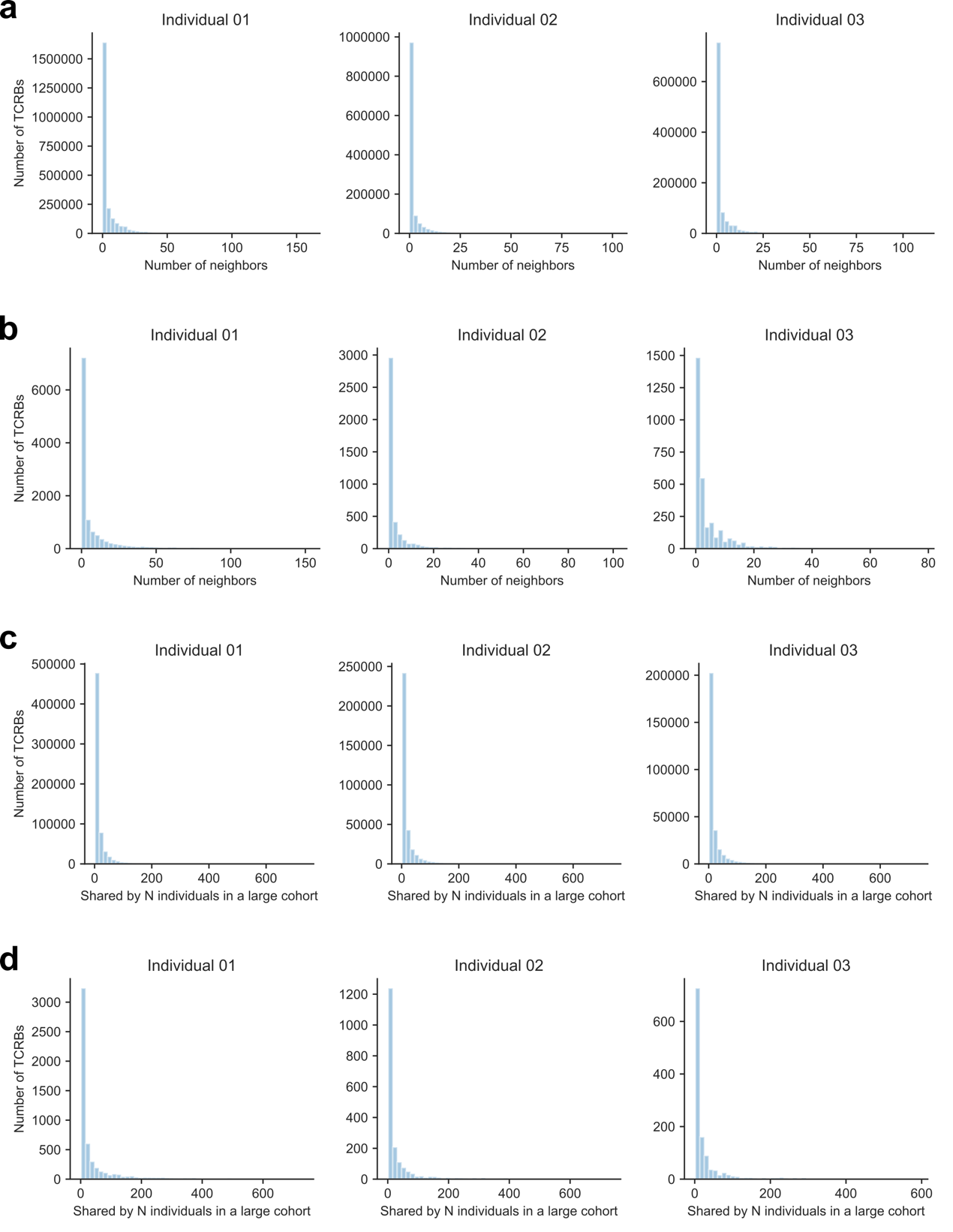


**Figure S13**. Distributions of the number of neighbors and degree of sharing across people for all TCRβs and high-abundance TCRβs. Each plot is a distribution of all TCRβs from PBMC samples for each individual (a, c) or only high-abundance TCRβs (b, d). Plots (a) and (b) show the number of neighbors in a network based on Levenshtein distance and plots (c) and (d) show the number of subjects sharing a given receptor in a large, independent, and similarly profiles cohort.


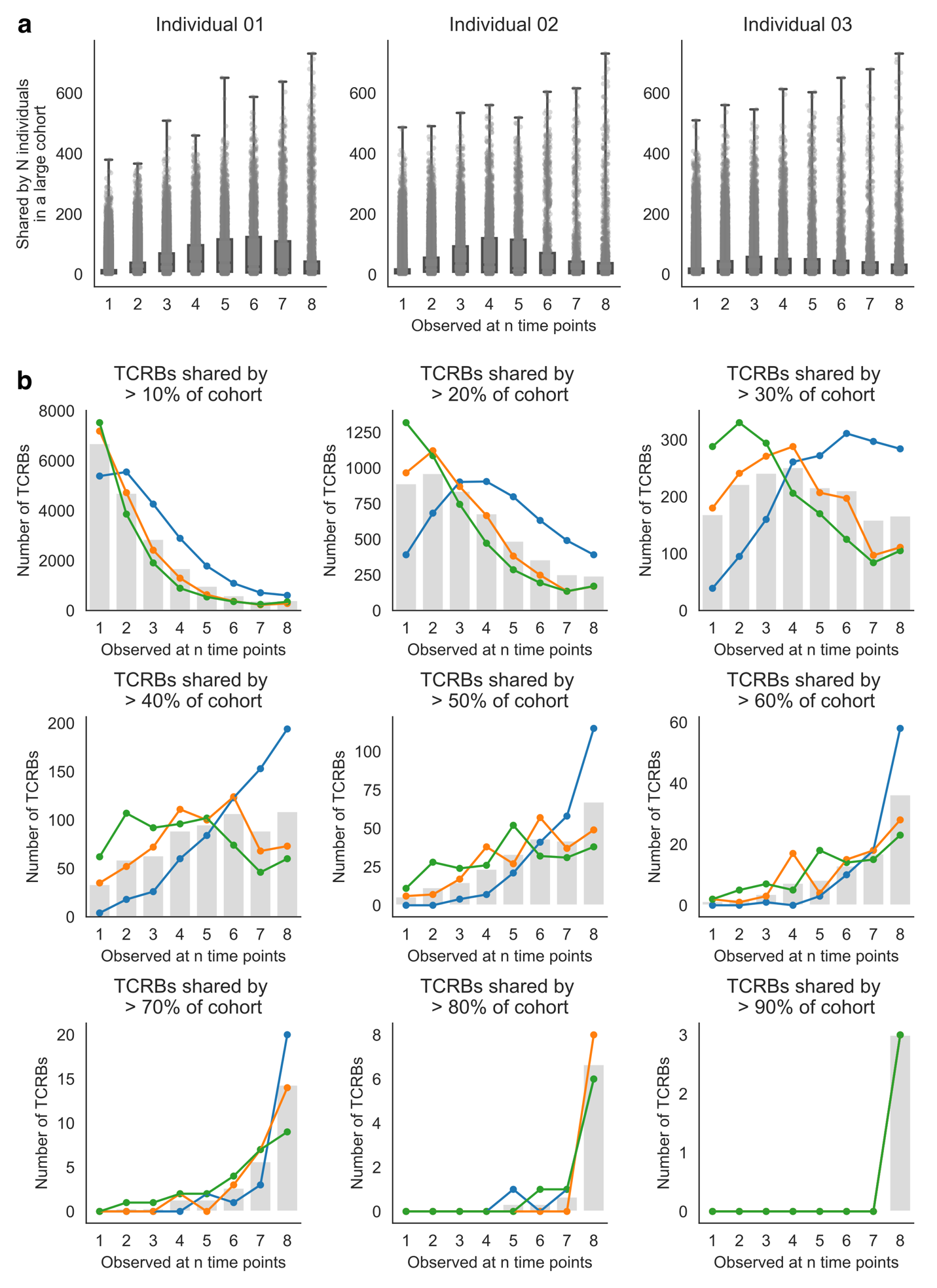


**Figure S14**. Persistent TCRβs were rich in highly public TCRβs. (a) Over all TCRβs, receptors that occurred at an intermediate number of time points were on average most-shared across people, but these distributions are heavily skewed toward private receptors. (b) We focused on TCRβs that were shared by at least a certain percentage of individuals in the large (*N* = 778) cohort. We found that less public TCRβs were generally observed at few time points, while highly public TCRβs were predominately observed at all time eight points. These results were even more striking given that we observed ~100–1000-fold more TCRβs occurring at a single time point than at all time points.

**
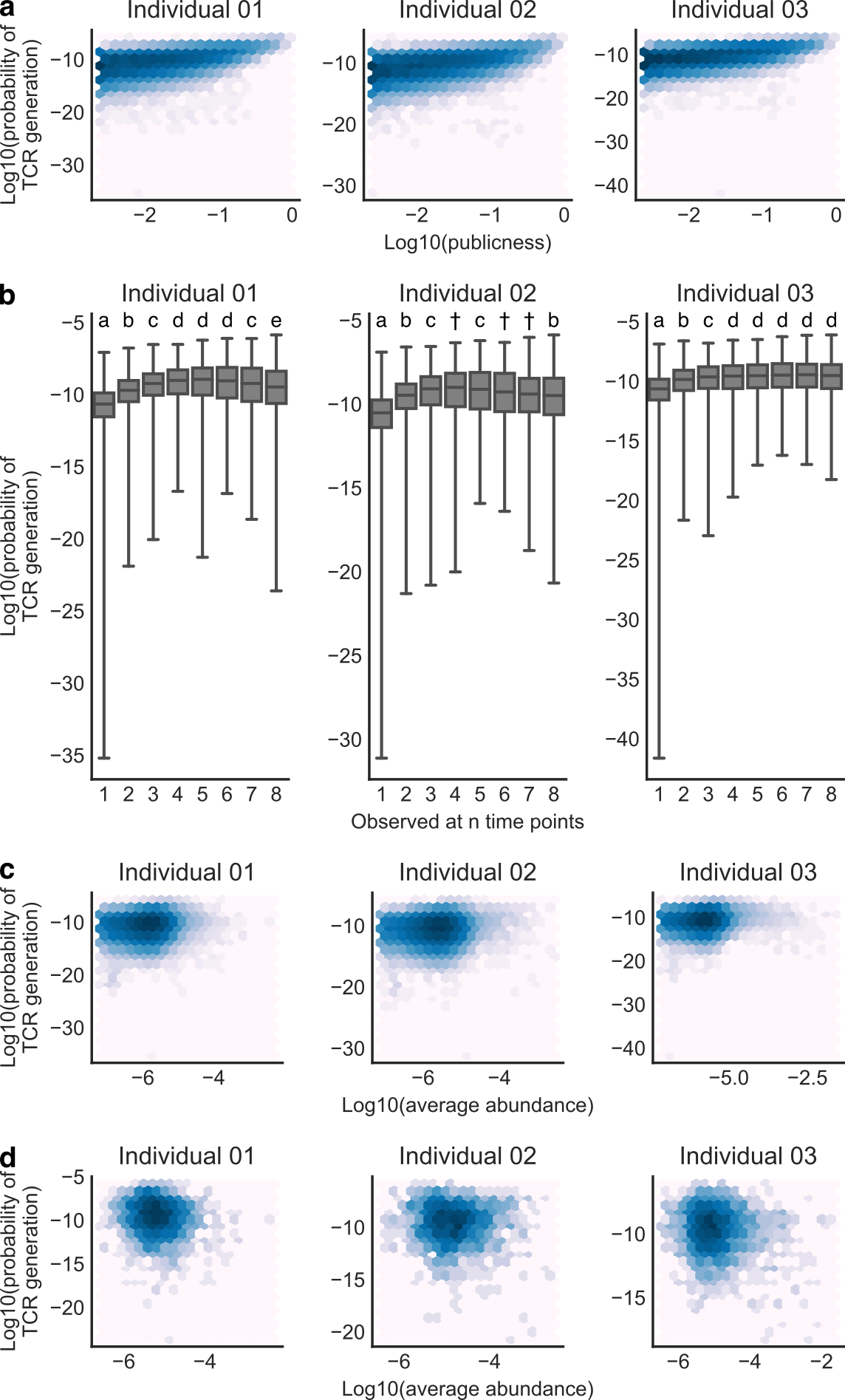
**

**Figure S15**. Persistent and public receptors may result in part from TCR recombination biases. (**a**) As in previous studies, the probability that a given TCRβ was generated correlated closely with publicness in a cohort of 778 individuals. For each individual, only TCRβs occurring in both that individual and the cohort were considered. (number of TCRβs evaluated in individual 01 = 638091, Spearman *rho* = 0.51111, *p* < 10^–6^; number of TCRβs evaluated in individual 02 = 338617, Spearman *rho* = 0.52231, *p* < 10^–6^; number of TCRβs evaluated in individual 03 = 284990, Spearman *rho* = 0.51129, *p* < 10^–6^). (**b**) TCRβs occurring at more time points tended to have higher generation probabilities, although persistent TCRβs did not have higher generation probabilities than other receptors observed at multiple time points. Letters indicate significant differences from all other groups by a Mann-Whitney *U* test (*p* < 0.001), while dagger (†) indicates groups that were not significantly different from multiple other groups. (**c**) Mean abundance of all TCRβs correlated significantly with generation probability but with a low correlation coefficient (individual 01: Spearman *rho* = 0.07884, *p* < 10^–6^; individual 02: Spearman *rho* = 0.05300, *p* < 10^–6^; individual 03: Spearman *rho* = 0.08208, *p* < 10^–6^). (**d**) Mean abundance of persistent TCRβs did not correlate with generation probability (persistent TCRβs: number of TCRβs evaluated in individual 01 = 3448, Spearman *rho* = –0.08988, *p* < 10^–6^; number of TCRβs evaluated in individual 02 = 1978, Spearman *rho* = –0.04341, *p* = 0.0537; number of TCRβs evaluated in individual 03 = 2965, Spearman *rho* = 0.04552, *p* = 0.01318)

Table S1. Overall TCRβ-sequencing statistics per sample: sequencing depth, productive TCRβ sequencing depth, fraction of productive TCRβ sequences, unique V genes identified, unique J genes identified, unique CDR3 sequences, unique TCRβs, unique TCRβ nucleotide sequences.

Table S2. V gene usage across subject and T cell population, expressed as both a fraction of all unique productive TCRβs and as a mean total abundance per sample.

Table S3. J gene usage across subject and T cell population, expressed as both a fraction of all unique productive TCRβs and as a mean total abundance per sample.

Table S4. Sequence and abundance information for the largest cohort of closely correlated TCRβs identified in each individual by Spearman’s or Pearson’s correlation.

Table S5. The fraction of TCRβs in each sample that occurred in 1–8 samples from that subject's time series.

Table S6. Mann-Whitney U test statistics for mean abundance of TCRβs occurring in different numbers of samples during the time series.

Table S7. Mann-Whitney U test statistics for nucleotide redundancy of TCRβs occurring in different numbers of samples during the time series.

Table S8. The fraction of TCRβs in each sample that were shared to different degrees among subjects in a large, independent cohort.
